# Aspirated Denture: A Rare Cause of Empyema in an Asian Male

**DOI:** 10.7759/cureus.17097

**Published:** 2021-08-11

**Authors:** Mustafa Bin Ali Zubairi, Maaha Ayub, Ali Zubairi, Kulsoom Fatima

**Affiliations:** 1 Medicine, Dow University of Health Sciences, Civil Hospital Karachi, Karachi, PAK; 2 Medicine Department, Aga Khan University Hospital, Karachi, PAK; 3 Pulmonology Department, Aga Khan University Hospital, Karachi, PAK; 4 Radiology Department, Aga Khan University Hospital, Karachi, PAK

**Keywords:** foreign body aspiration, denture, empyema, pneumonia, ct chest

## Abstract

Here, we report the case of an undiagnosed foreign body aspiration (FBA) in a 50-year-old male who presented with fever, productive cough, and shortness of breath suggestive of pneumonia. The patient reported a history of empyema for which he underwent left-sided video-assisted thoracoscopic surgery and decortication at another facility. Careful evaluation of prior chest imaging revealed a radio-opaque linear density projecting along the left of his spine suspicious for a foreign body in the airway which was missed on radiographic evaluation at the time of empyema. On flexible fiberoptic bronchoscopy, an irretrievable foreign body was visualized in the patient’s left mainstem bronchus, which was removed via rigid bronchoscopy. This is a rare case of an aspirated denture manifesting as empyema and subsequently as pneumonia. We suggest that in patients with a recurrent chest infection, the possibility of FBA must be ruled out by detailed history and careful evaluation of imaging.

## Introduction

Foreign body aspiration (FBA) is an obstruction of the respiratory pathway owing to the presence of a liquid or solid entity [1]. It can lead to major complications and is associated with a significant rate of morbidity and mortality [2,3]. Risk factors for FBA include mental retardation, maxillofacial trauma, unconsciousness, intoxication, dementia, use of sedative drugs, and dental prosthesis [2]. Unfortunately, these patients present with nonspecific signs and symptoms which may delay the diagnosis [1].

In this case report, we discuss a patient who presented with FBA manifesting as empyema. Although the patient was initially managed at another facility, the FBA remained undiagnosed for a year.

This case report was previously presented as a poster abstract at the CHEST Annual Virtual Meeting held from October 18 to October 21, 2020, in Chicago.

## Case presentation

A 50-year-old diabetic male presented to the emergency department at the Aga Khan University Hospital, Karachi with complaints of fever, productive cough, and shortness of breath for two weeks. The patient was a betel quid addict (a form of chewing tobacco known as gutka in Southeast Asia) and an ex-smoker with a five-pack-year smoking history; however, he had stopped smoking since the last five years. His past medical history was significant for empyema, for which he underwent video-assisted thoracoscopic surgery (VATS) and decortication a year ago at another facility, followed by treatment with intravenous (IV) piperacillin/tazobactam and ciprofloxacin, and had been discharged in a stable condition.

**Figure 1:**
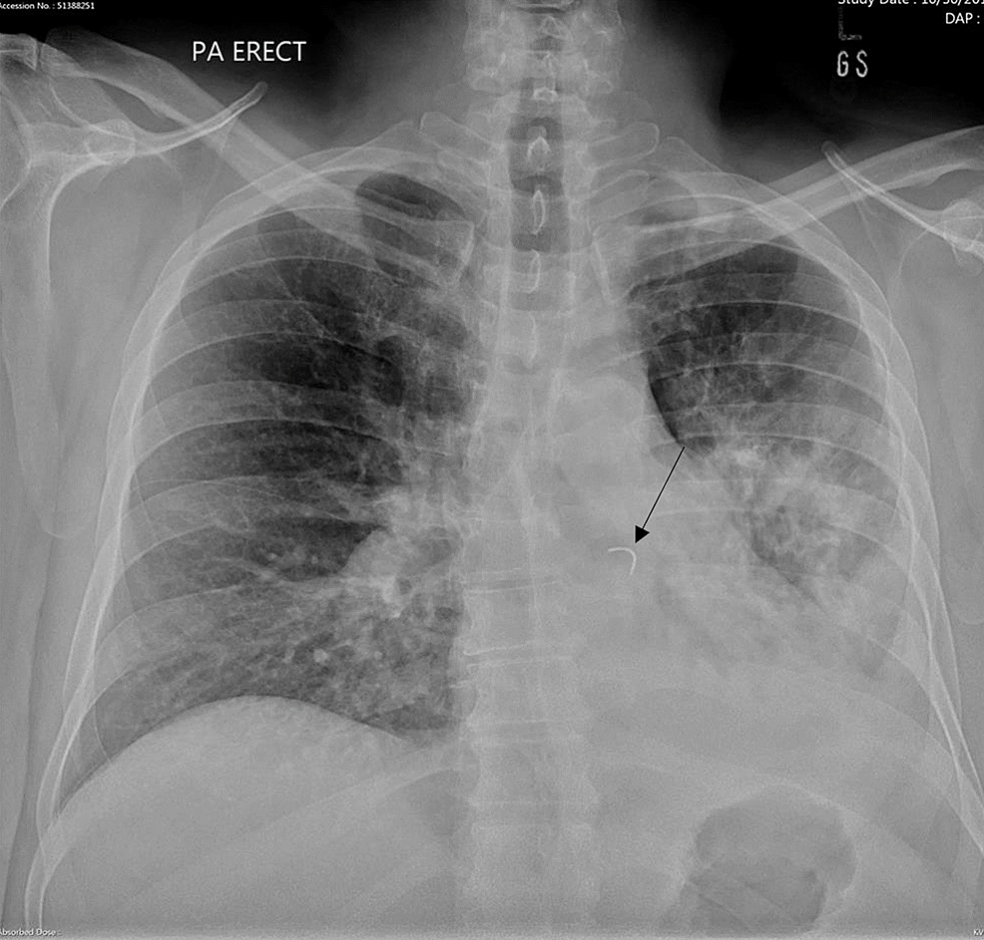
PA view of the chest X-ray chest. A curved radio-opaque density is seen projecting over the left lower lobe bronchus (black arrow). Inhomogeneous shadowing in the left mid and lower lung zone with air bronchogram representing consolidation and obscured left hemidiaphragm and left costophrenic angle suggesting mild left pleural effusion. PA: posteroanterior

**Figure 2:**
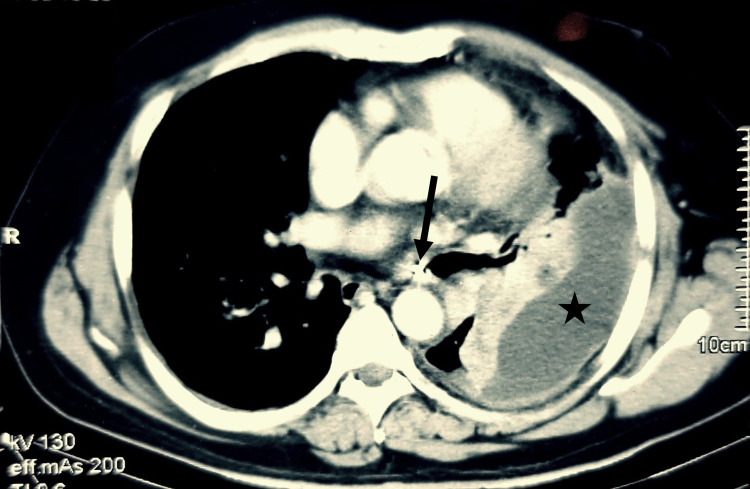
Axial CT scan (mediastinal window) performed one year previously. The metallic density with streak artifacts noted within the left main bronchus (black arrow). Left-sided empyema (black star) with adjacent left lower lobe atelectasis. CT: computed tomography

**Figure 3:**
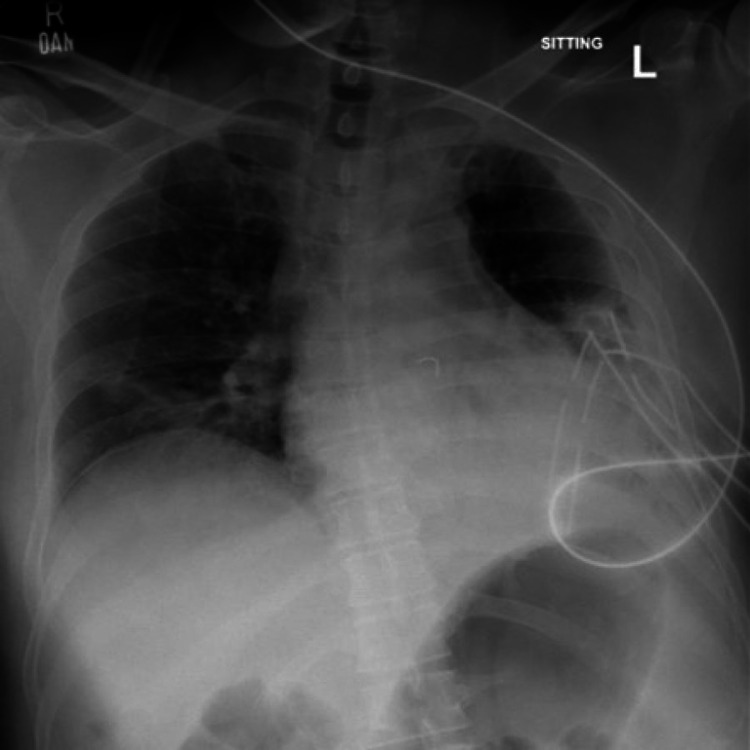
Post-VATS chest X-ray performed a year ago. Left-sided chest tubes are in place and empyema has resolved. The subtle radio-opaque density is shown by a black arrow. VATS: video-assisted thoracoscopic surgery

On examination, he had a pulse of 76 beats per minute, temperature of 37.8°C, blood pressure of 134/76 mmHg, respiratory rate of 22 breaths per minute, and oxygen saturation of 98%. On chest examination, bilateral lung crackles were audible along with decreased breath sounds in the left lower lung zone. The rest of the examination was unremarkable. His complete blood count revealed a hemoglobin of 11 g/dL (normal: 12.3-16.6 g/dL), hematocrit of 34.5% (normal: 38.4-50.7%), white blood cell count of 13.8 × 10^9^/L (normal: 4.8-11.3 × 10^9^/L), and platelets of 425 × 10^9^/L (normal: 154-433 × 10^9^/L). Gram stain and acid-fast bacillus smear of sputum were negative. Chest X-ray (CXR) revealed left mid and lower lung zone infiltrates with probable mild left-sided pleural effusion and a curved radio-opaque density projecting over the left lower lobe bronchus (Figure 1). A careful review of his previous chest CT before VATS (Figure 2) and his post-VATS CXR (Figure 3), both done a year earlier at another facility, demonstrated a metallic density within the left main bronchus, consistent with the current CXR findings.

**Figure 4:**
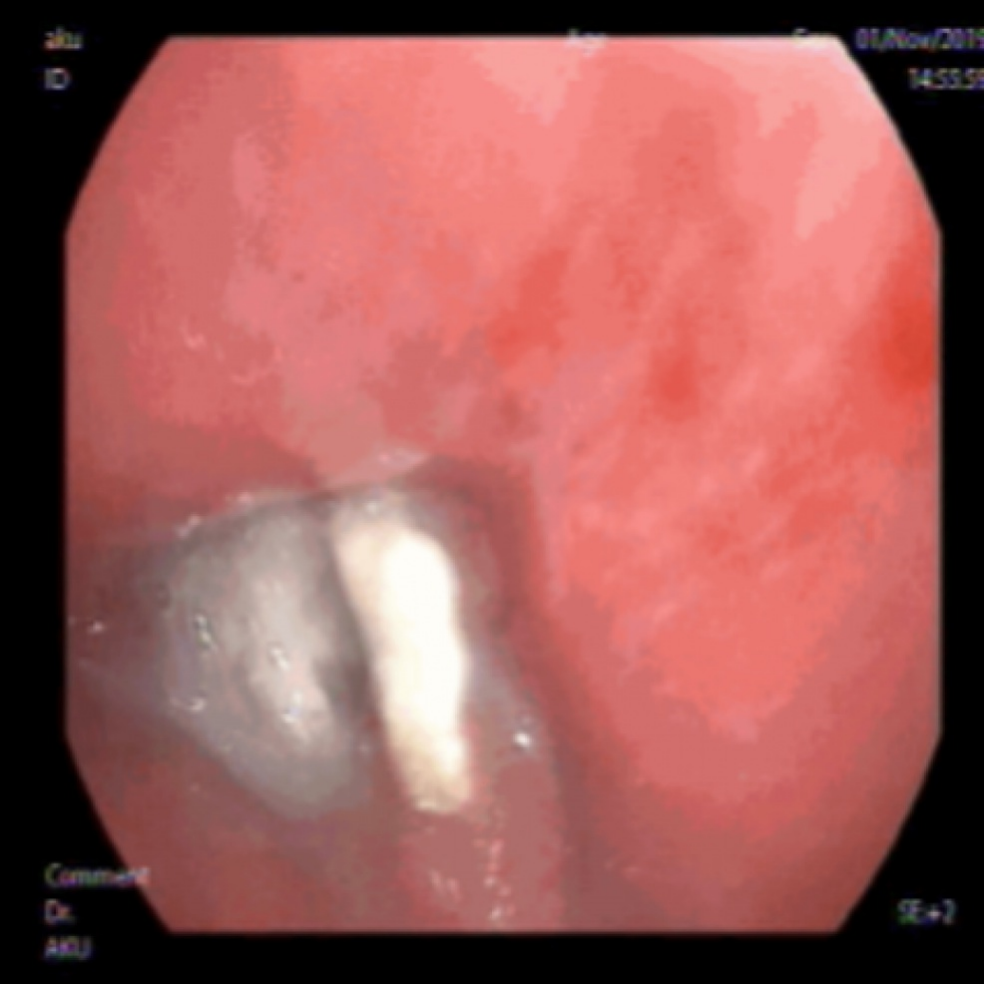
Foreign body visualized on flexible fiberoptic bronchoscopy.

**Figure 5:**
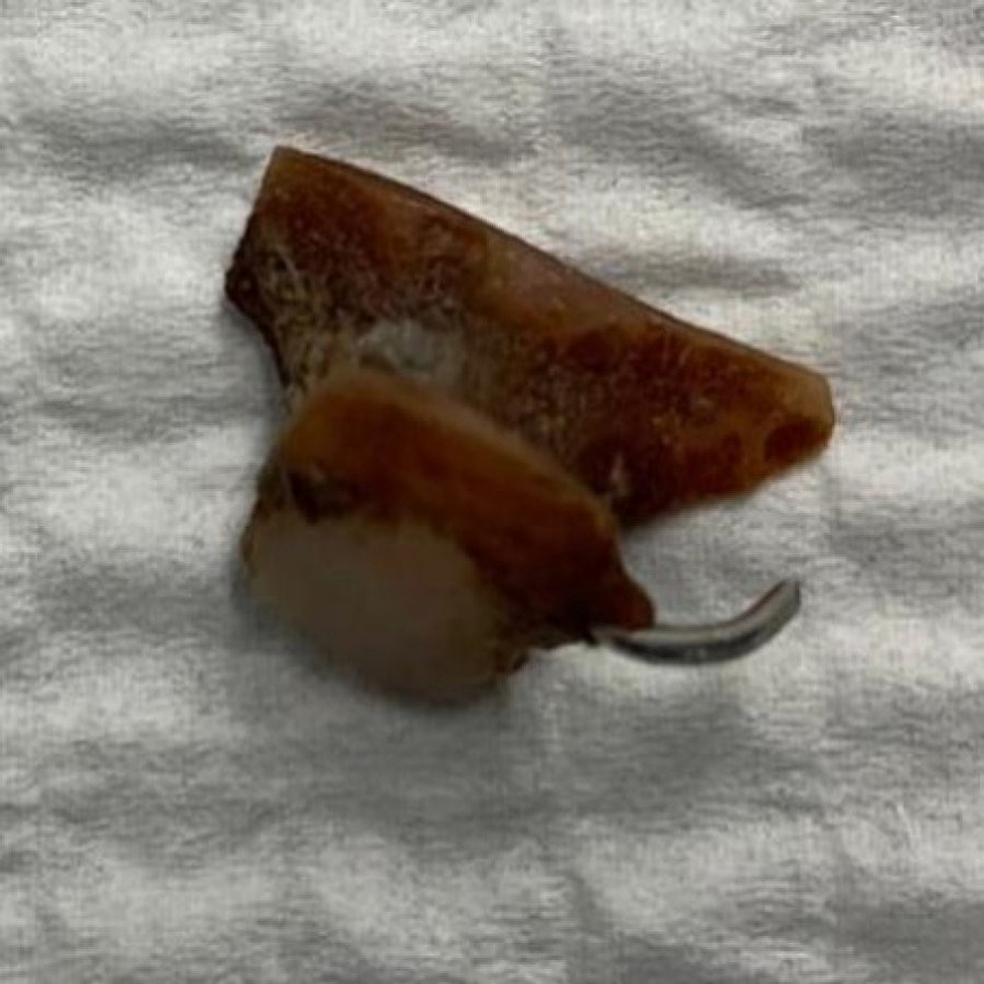
The denture retrieved via rigid bronchoscopy.

He was started on IV ceftriaxone and oral azithromycin. Flexible fiberoptic bronchoscopy was performed which revealed an irretrievable foreign body in the left mainstem bronchus (Figure 4). The foreign body was successfully removed by rigid bronchoscopy and revealed to be a denture (Figure 5). On further inquiry, the patient disclosed that he had misplaced his denture a year ago, before his hospital admission at another facility, not realizing that he had swallowed them while sleeping.

His condition subsequently improved, and he was discharged on oral amoxicillin/clavulanic acid and ciprofloxacin. Upon follow-up in the clinic seven days later, the patient’s condition was stable and his antibiotics were discontinued. He remained well during subsequent follow-up visits.

## Discussion

FBA is common in young children and the elderly but relatively uncommon in the adult population [4,5]. Aspirated foreign bodies most commonly become lodged in the right mainstem bronchus [1]. This is attributed to the anatomical positioning of the right mainstem bronchus, which makes it easier for foreign bodies to lodge [3]. On the contrary, our patient had a foreign body lodged in his left mainstem bronchus which may be attributed to his positioning during sleep.

A few cases of aspirated dentures have been reported in the literature [6,7]; however, these patients had predisposing factors. Risk factors for FBA include mental retardation, maxillofacial trauma, unconsciousness, intoxication, dementia, use of sedative drugs, and dental prosthesis [2]. Haghighi et al. [6] described the case of a 90-year-old man who presented with upper denture aspiration, while Kent et al. [7] described a case of denture aspiration following maxillofacial trauma. In our case, there was no known predisposing condition that could have led to aspiration.

Adults with FBA normally present with a wide range of nonspecific symptoms such as cough, hemoptysis, dyspnea, and fever. However, often, FBA may remain asymptomatic [1]. Approximately, 80% of the aspirated foreign bodies cannot be visualized on CXR because only 10% are radio-opaque [8,9]. CT scan can depict a foreign body as an intrabronchial or intraparenchymal mass [10]. Although the aspirated foreign body in our case was a radio-opaque denture, it was missed on the initial CXR and CT scan performed at an outside facility.

FBA can lead to multiple respiratory complications such as pneumomediastinum, pneumothorax, hydropneumothorax, bronchial stenosis, lung abscess, atelectasis, pneumonia, bronchiectasis, foreign body dislodgment, and bronchospasm [11]. However, empyema is extremely rare with only one case reported previously [12].

Flexible bronchoscopy is effective and safe for foreign body retrieval among adults. Sehgal et al. [4] reported a 92% success rate for flexible bronchoscopy. Flexible bronchoscopy is simple and can be performed as an outpatient procedure without administering general anesthesia. It is, therefore, associated with a lower risk of morbidity and mortality compared to rigid bronchoscopy. However, in few situations, where the foreign body is irretrievable by flexible bronchoscopy, as in our case, rigid bronchoscopy is indicated.

## Conclusions

FBA often presents with nonspecific symptoms causing difficulty in diagnosis and can lead to multiple complications. Aspiration of dentures among middle-aged people with no known predisposing factor for aspiration is a rare occurrence. A thorough history and careful radiological evaluation are essential for timely detection of FBA and for preventing subsequent complications.
